# Know Thyself: NK-Cell Inhibitory Receptors Prompt Self-Tolerance, Education, and Viral Control

**DOI:** 10.3389/fimmu.2014.00175

**Published:** 2014-04-16

**Authors:** William T. Nash, Jeffrey Teoh, Hairong Wei, Awndre Gamache, Michael G. Brown

**Affiliations:** ^1^Department of Microbiology, Immunology, and Cancer Biology, School of Medicine, University of Virginia, Charlottesville, VA, USA; ^2^Beirne B. Carter Center for Immunology Research, School of Medicine, University of Virginia, Charlottesville, VA, USA; ^3^Division of Nephrology, Department of Medicine, University of Virginia, Charlottesville, VA, USA

**Keywords:** NK cells, inhibitory receptors, virus control, immunity, education, licensing

## Abstract

Natural killer (NK) cells provide essential protection against viral infections. One of the defining features of this lymphocyte population is the expression of a wide array of variable cell surface stimulatory and inhibitory NK receptors (sNKR and iNKR, respectively). The iNKR are particularly important in terms of NK-cell education. As receptors specific for MHC class I (MHC I) molecules, they are responsible for self-tolerance and adjusting NK-cell reactivity based on the expression level of self-MHC I. The end result of this education is twofold: (1) inhibitory signaling tunes the functional capacity of the NK cell, endowing greater potency with greater education, and (2) education on self allows the NK cell to detect aberrations in MHC I expression, a common occurrence during many viral infections. Many studies have indicated an important role for iNKR and MHC I in disease, making these receptors attractive targets for manipulating NK-cell reactivity in the clinic. A greater understanding of iNKR and their ability to regulate NK cells will provide a basis for future attempts at translating their potential utility into benefits for human health.

## Introduction

Natural killer (NK) cells are innate lymphocytes with broad specificity and a capacity for rapid recognition and activation. They participate in a wide variety of biological conditions spanning cancer, autoimmunity, pregnancy, and viral infections. Although originally identified and named for their ability to spontaneously lyse tumor cells (1, 2), clear evidence showcasing their importance comes from studies of virus control in the context of NK-cell deficiency. Human classical NK deficiency results in reduced numbers of NK cells, while functional NK deficiency results in normal NK-cell numbers but impaired responsiveness (3). Individuals exhibiting either of these conditions consistently present in the clinic with severe viral infections – especially herpesviruses (3, 4).

Unlike T and B cells, NK cells do not undergo DNA rearrangements to generate their cell surface receptors. Rather, they express a broad array of stimulatory and inhibitory NK receptors (NKR) encoded by genes clustered in the NK gene complex (NKC) and leukocyte receptor complex (LRC) (5–11). NK cells in both mice and humans express NKC-encoded C-type lectin-like receptors including NKG2D, NKG2A/C/E, and CD94. Mouse NK cells also express diverse Ly49 receptors, another NKC-encoded family of C-type lectin-like receptors, whereas human NK cells express variable LRC-encoded killer Ig-like receptors (KIRs) (5–11).

The NKC and LRC represent two of the most variable and diverse genomic intervals, rivaling the MHC in terms of gene content (7, 9, 12). Diversification of genomic intervals encoding NKR is evident in many species (7, 9) and is likely shaped by a variety of factors influencing fitness and survival, including pathogen resistance, detection of self-MHC I ligands, and reproductive success (10). Two main points related to NKR variability require emphasis: (1) Independent convergent evolution of the NKR in many species highlights their functional importance; and (2) extensive intra- and inter-species NKR gene diversity is indicative of their instability and rapid evolution, likely due to balancing selection exerted by both MHC and pathogen genetics (9).

NK receptor expression in NK cells is variegated and can vary greatly from NK cell to NK cell. Interactions between NKR and their environment establish a balance of signals within the NK cell, favoring either tolerance or activation (12). Inhibitory (i)NKR specifically bind MHC class I molecules as ligands, allowing NK cells to patrol for normal self-MHC expression. Stimulatory (s)NKR bind to a variety of ligands including viral proteins and MHC I-related molecules induced by cellular stress pathways (12–14). While roles for a number of sNKR in NK-mediated virus control have been established (15–17), the contributions of iNKR have been more difficult to unravel. Specific detection of viral or virus-induced host ligands by sNKR has been shown to enhance NK-cell activation, expansion, and viral clearance. However, a large body of work in the field also links iNKR activity to NK-cell target detection, sensitivity to sNKR stimulation, and capacity for effector function (cytokine production and target lysis). Thus, iNKR are likely to have prominent effects on immune responses, extending well beyond the disruption of activation signals and self-tolerance.

Here, we consider data concerning iNKR involvement in immunity. The discussion focuses on the importance of iNKR activity in a variety of processes including driving NK functional tuning, shaping viral evasion strategies, enhancing activation responses, and mediating specific recognition and control of viral infections.

## Inhibitory Receptors Increase NK-Cell Responsiveness

As mediators of NK-cell self-tolerance, iNKR safeguard against aberrant or chronic immune activation and the development of autoimmunity (18–24). However, iNKR signaling also leads to increased basal responsiveness in NK cells (25–30). Cognate interactions between iNKR and self-MHC I ligands tune the responsiveness of NK cells. This occurs through an educational process that has been referred to as “arming” or “licensing.” When NK cells express an iNKR that can bind at least one self-MHC I ligand in their environment, they are said to be “licensed.” These NK cells display increased *ex vivo* sensitivity to stimulatory receptor cross-linking compared to “unlicensed” NK cells lacking self-specific iNKR.

**Figure 1:**
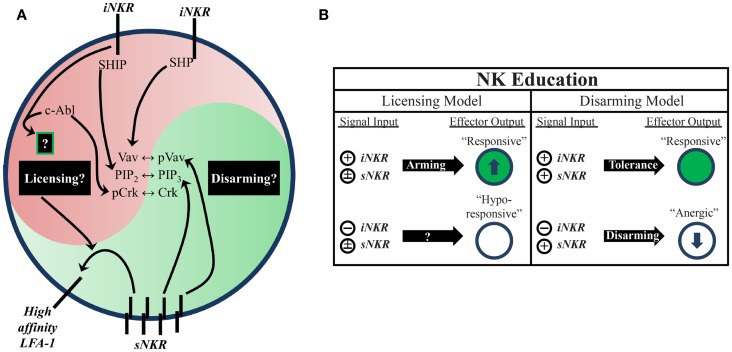
**Natural killer education primes NK cells for heightened effector function**. **(A)** Inhibitory signaling serves a twofold purpose. On one hand, it can disrupt activation signals from sNKR at several intersections (e.g., SHP dephosphorylation of Vav, SHIP dephosphorylation of PIP_3_, and c-Abl sequestration of Crk from activation complexes). On the other hand, it also serves to tune the reactivity of the NK cell to activating stimuli, either through unknown positive signals transmitted downstream of iNKR ligation (licensing model) or prevention of anergy (disarming model). One distinct benefit of self-specific iNKR that has been recently established is the ability to enhance sNKR inside-out signaling to LFA-1 to promote adhesion and target recognition. **(B)** The balance of signals in NK cells determines their reactivity. NK that do not receive inhibitory signals can be activated in response to inflammatory stimuli and conditions, but are generally less responsive to sNKR stimulation (in terms of cytokine production and cytotoxicity) than NK that receive iNKR input. Whether this occurs via a licensing mechanism, disarming mechanism, or both is still not fully worked out.

Two proposed models attempt to account for the differential responsiveness of NK cells stemming from the presence or absence of self-MHC binding iNKR. The “disarming” model contends that NK cells without any iNKR for self-MHC I are rendered hyporesponsive due to chronic low-level stimulation; whereas the “licensing” model predicts that NK cells without iNKR for self-MHC I simply fail to acquire full reactivity (Figure 1) (26, 31). Adherence to one or the other of these hypotheses may be too idealistic, though, as there is evidence to support both and they may indeed be occurring side-by-side in NK cells. Regardless of the mechanism, NK cells that sense self at steady state are more reactive to stimulation and changes in MHC class I expression than their self-ignorant counterparts.

Such iNKR licensing effectively bestows an added level of sensitivity to self-MHC ligand expression. NK tuning to normal levels of self-MHC expression broadens NK-cell specificity, allowing licensed-NK cells to detect and respond against cellular targets failing to express adequate levels of self-ligand (17, 32, 33). In short, productive licensing through inhibitory signaling provides a twofold benefit to NK function. It serves to simultaneously enhance effector responses (e.g., IFNγ secretion and cytotoxicity) and broaden the NK-cell’s target specificity to include aberrant cells that would not be detected by stimulatory receptors alone.

In light of these advantages, it is important to emphasize that licensing is a tunable process, i.e., that the extent of inhibitory receptor priming corresponds to the relative increase in NK-cell reactivity (34–36). Hence, the licensing effect is not a binary readout. Instead, it manifests as a rheostat determined by the total input from iNKR. Whether the enhancement of NK responsiveness is actively mediated by iNKR signals or simply the result of increased disruption of stimulatory NKR signaling is an important question that has yet to be resolved. Moreover, the licensing status of an NK cell is not fixed. Rather, several studies have shown that the responsiveness of NK cells can be reset after their transfer from one MHC I environment to another (37–39). These results suggest that the acquisition of functional reactivity is a dynamic process that results in continual NK-cell re-education.

While the mechanistic basis of NK-cell education is still unclear, it is known that the immunoreceptor tyrosine-based inhibitory motif (ITIM) in the iNKR cytoplasmic tail is required (29). Both mouse and human iNKR carry at least one ITIM that can bind cellular phosphatases containing Src homology 2 (SH2) domains. Phosphatase recruitment further leads to Vav dephosphorylation and the subsequent disruption of activation signals (Figure 1) (17). The role of SH2-containing phosphatases in NK-cell licensing and function has also been the subject of investigation (40, 41). However, results from these studies have been difficult to interpret. The effects of SH2-containing tyrosine phosphatase 2 (SHP-2) on licensing are not well known due to the embryonic lethality of knockout mice (42). SH2-containing inositol phosphatase (SHIP) deficient mice exhibit skewed NKR repertoires with increased representation of certain iNKR (43). NK cells in these mice appear to have a defect in the enhancement of IFNγ production but have intact missing-self recognition in response to reduced iNKR ligation (43, 44). SHP-1 has been studied in more detail than SHP-2 and is reported as the most prevalent phosphatase recruited to ITIMs during MHC-specific iNKR inhibition (25). In contrast with SHIP−/−, SHP-1 deficient mice had intact iNKR-mediated enhancement of IFNγ but a markedly reduced ability to lyse targets with reduced or absent MHC I expression (29, 45). These results highlight the complexity of NK-cell education and may suggest that iNKR license NK cells via multiple, distinct pathways.

Indeed, there is now evidence for direct signaling events downstream from inhibitory receptors (17, 46, 47). Rather than inducing complete broad-scale dephosphorylation, inhibitory receptor signaling also leads to specific phosphorylation of the small adaptor molecule, Crk (46). This results in its association with c-Abl and sequestration from activation signaling complexes (47). While it is still unknown if this leads to other downstream effects that may result from inhibitory receptor cross-linking, this discovery opens the possibility that inhibitory signaling could induce additional phosphorylation events that have yet to be identified (Figure 1).

Increased target cell adherence during conjugate formation is another positive effect of NK-cell education (48). Strong LFA-1-dependent adhesion induces the formation of tight conjugates between NK cells and target cells (49, 50), as well as the subsequent polarization of lytic granules (51). Inside-out signaling from stimulatory receptors to LFA-1 causes it to shift into an open conformation, allowing for a stronger association with ICAM on target cells (52). Interestingly, cells educated via inhibitory signals were found to have enhanced activating receptor inside-out signaling (Figure 1). Following incubation with K562 target cells, activation signals in licensed-NK cells resulted in more high-affinity (open conformation) LFA-1 when compared to their unlicensed counterparts, which resulted in increased tight conjugate formation, lytic granule polarization, and target cell lysis (48). Thus, inhibitory tuning of NK cells potentiates increased responsiveness by enhancing sNKR signaling. Although the mechanistic basis of this effect on sNKR signaling is unknown, we can infer that iNKR signaling pathways exert a profound influence on the reactivity of licensed-NK cells.

Collectively, these studies provide substantial evidence of a critical role for iNKR signaling in the tuning of NK-cell function. While unlicensed-NK cells are capable effectors, education via iNKR provides NK cells with enhanced activation potential and an added layer of specificity for the detection of MHC class I fluctuations and/or aberrations. Such tuning is essential for the role of NK cells as sentinels of healthy self-gene expression.

## Viral Evasion Tactics Underscore iNKR Immune Pressure

**Figure 2:**
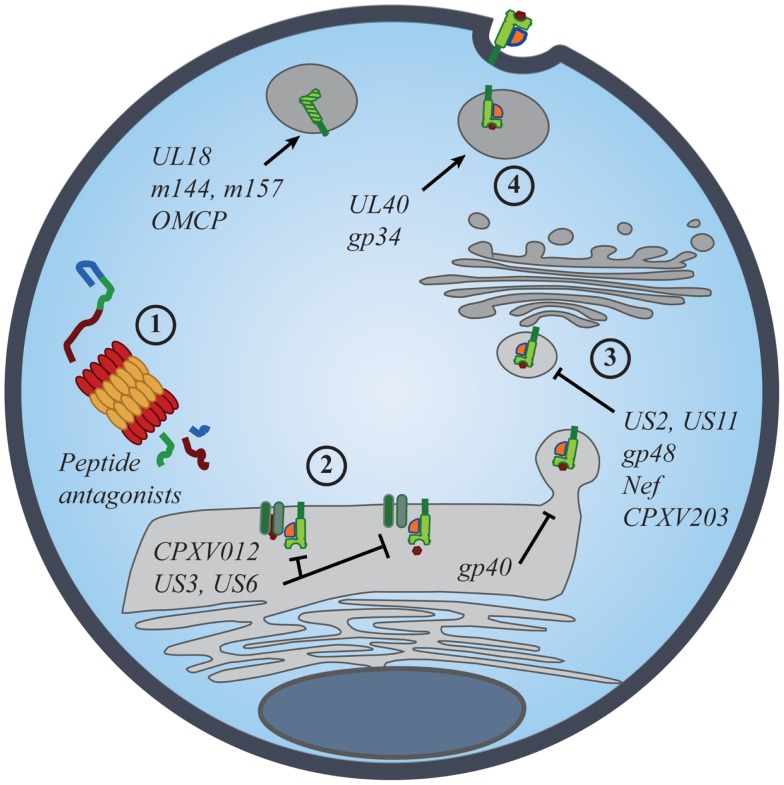
**Viral manipulation of the MHC class I antigen processing and presentation**. The presentation of self- and foreign-peptides on MHC class I molecules is integral to the regulation of both NK and CD8+ T cell immunity. Viruses have developed multiple strategies to interfere with class I antigen presentation, with the ultimate goal of evading both innate and adaptive immune recognition. Manipulation of class I molecules can occur at various stages of the class I expression pathway: (1) peptide antagonists produced during proteasomal degradation of cytoplasmic proteins dictate the affinity of class I interactions with iNKR and CD8+ TCR; (2) immunoevasins interfere with the proper loading and folding of the class I molecules, and even retain properly folded molecules in the ER; (3) viral proteins promote the export of class I molecules into the cytosol for proteasomal degradation or re-direct class I trafficking from the ER to endo-lysosomal compartments; (4) viral proteins promote the expression of classical and non-classical MHC molecules, as well as class I mimics, to specifically inhibit NK-cell activation.

Evidence for the importance of NK-cell iNKR in viral resistance is seen in the immune evasion mechanisms employed by viruses. Common viral strategies to avoid detection by CD8+ T cells involve the regulation of MHC I expression on the surface of infected cells. However, viruses must strike a balance between limiting MHC I presentation of virus-derived peptides and maintaining sufficient MHC I levels to prevent “missing-self” detection. Such manipulations have been described for many viruses, particularly those most susceptible to NK-mediated responses [Figure 2; reviewed in Ref. (53, 54)]. While many viruses exploit iNKR as a way to evade immune detection, the targeted nature of these diverse strategies conveys the prominence of selective pressure exerted by NK cells and their iNKR.

### Selective modulation of MHC I to inhibit NK cells

Human (H)CMV utilizes several immunoevasin proteins to selectively decrease MHC I expression on the surface of infected cells. Although seemingly redundant, the varied timing of expression, mechanisms of action, and allelic-specificities of these immunoevasins indicate a tightly orchestrated manipulation of class I expression responsible for HCMV’s successful evasion of the immune system. Both US3 and US6 retain MHC I in the ER. US3 interferes with tapasin-dependent peptide antigen transport (55–57), whereas US6 binds to TAP and prevents the proper loading and folding of all MHC I (58, 59). As neither of these immunoevasins directly binds to specific class I alleles, their class I targets for downregulation are quite broad. US2 and US11, on the other hand, target and bind to specific, non-overlapping alleles of HLA-A and -B molecules for dislocation from the Golgi apparatus and subsequent degradation in the cytosol, leaving residual HLA-C and -E expression to bind inhibitory KIR and NKG2A/CD94, respectively (59–61). Indeed, the non-overlapping specificities of these latter immunoevasins are indicative of the virus’s acquired responses to the high degree of MHC I polymorphism (62). However, it is also possible that this arsenal of independent immunoevasins and their regulation was driven by iNKR. By encoding for non-redundant manipulators of class I expression, HCMV selectively regulates MHC surface expression in an attempt to protect itself from missing-self detection by NK cells.

Similar to HCMV, HIV-1 and cowpox virus (CPXV) downregulate surface expression of MHC I molecules to avoid lysis by CD8+ T cells. HIV Nef binds the cytoplasmic tails of HLA-A and -B molecules in the ER and then redirects them to endo-lysosomal compartments for degradation (63). Minor differences in the tails of HLA-C and -E prevent Nef from interfering with their trafficking to the cell surface, which in turn renders infected CD4+ T cells resistant to lysis by NK cells expressing the cognate inhibitory KIR (64–66). In cases where HLA-B is the cognate-ligand for inhibitory KIR, Nef-mediated downregulation of MHC I ligands may actually enhance NK-cell control of HIV-1 infection (discussed below) (67–69). However, pressures from self-licensed-NK cells may also select against Nef-mediated downregulation of HLA-B, as HLA-B appears to be more resistant to binding by certain Nef variants than HLA-A (70). CPXV proteins CPXV012 and CPXV203 diminish MHC I expression by blocking peptide transport into the ER and then interfering with MHC I trafficking in the Golgi (71–74). Further study is warranted to investigate how MHC I interference relates to HIV and CPXV evasion of NK-cell attack and the effect of host MHC polymorphism on NK and T cell recognition of virus-infected targets.

In addition to selective downregulation of allele-specific class I ligands, viruses also induce MHC I expression to inhibit NK cells. For example, a nonamer peptide derived from the leader sequence of HCMV UL40 that resembles the signal peptides of many HLA-C allotypes, binds to non-classical HLA-E in a TAP-independent manner (75, 76). This facilitates HLA-E export to the cell surface where it can engage the NKG2A/CD94 iNKR on NK cells. The promotion of inhibitory ligands by HCMV is beneficial and perhaps even necessary for immune evasion, as sequestration and degradation of classical MHC I molecules prevents the loading of signal peptides onto HLA-E, leaving the cell surface largely bare of MHC. However, increased expression of HLA-E can also render infected cells more susceptible to NK cells bearing the NKG2C/CD94 sNKR. A recent study of UL40 comparing clinical isolates of HCMV to the AD169 laboratory strain revealed increased polymorphism in the leader sequence mimic region from several isolates (77). While these polymorphisms had little impact on the ability of UL40-derived signal peptides to bind to and express HLA-E at the cell surface, non-conservative amino acid variations at specific positions were capable of abrogating NKG2C/CD94-mediated lysis while preserving inhibition through NKG2A/CD94.

Murine (M)CMV also encodes several glycoproteins that modify surface expression of MHC I molecules. MCMV gp40 sequesters loaded MHC I molecules in ER–Golgi intermediate compartments (78), whereas gp48 re-routes mature MHC I molecules to endo-lysosomal compartments for degradation (79). To balance this broad class I downregulation, MCMV gp34 binds and promotes MHC I expression at the cell surface (80–82). Indeed, these immunoevasins are both cooperative and antagonistic in function, and through a hierarchy of allele-specific binding, MCMV regulates surface MHC I expression to evade detection by NK cells and CD8+ T cells (83–85). Interestingly, gp34 mutations were found to accumulate over time in MCMV strains (86, 87), potentially allowing rapid adaptation of its MHC binding specificity. Thus, MCMV utilizes multiple strategies to fine-tune MHC I expression and evasion of iNKR+ cells.

Viral microRNAs also have a role to play in evading the immune system (88). Interestingly, work so far on the subject seems to suggest a major role for these viral miRNAs in regulating the expression of certain ligands of the major sNKR NKG2D (89). For example, at least three separate herpesviruses have been shown to produce distinct miRNAs that all target the stress-inducible NKG2D ligand MICB. HCMV, Kaposi’s sarcoma-associated herpesvirus (KSHV), and Epstein–Barr virus (EBV) all encode their own miRNA (miR-UL112, miR-K12-7, and miR-BART2-5p, respectively) that bind adjacent regions of the MICB 3′ UTR and interfere with its translation and expression (90, 91). It is interesting to note that this mechanism of NK evasion favors reduced sNKR recognition over increased iNKR inhibition; so there is not necessarily a direct impact on iNKR signaling. This shows that viruses exploit the internal regulation of NK cells (i.e., the “balance of signals”) either by increasing inhibition or reducing stimulation to escape NK detection. It is tempting to speculate here that a licensed NK may have an advantage in the face of MICB downregulation since they are more sensitive to activation stimuli, however this has not yet been explored.

## Evasion of NK Cells Though Molecular Mimicry

On top of selective editing of the class I repertoire, viruses use MHC I-related protein mimics that directly interfere with NK cells. The HCMV glycoprotein UL18, for example, binds to the iNKR ILT2/LIR-1 to interfere with LIR1+ NK-cell mediated cytotoxicity (92). For LIR1+ NK cells, the degree of UL18-mediated inhibition was directly related to the amount of LIR-1 available on the NK-cell surface. Interestingly, while highly protective against LIR1+ cells on a clonal level, UL18+ target cells actually increased the cytotoxicity of LIR1− NK cells through LIR-1-independent mechanisms. In a polyclonal population of NK cells, this increased cytotoxicity of LIR-1-cells was generally sufficient to mask the inhibition afforded by UL18. CPXV OMCP is another MHC I-related molecule that may interfere with NK cells by antagonizing NKG2D-mediated NK-cell stimulation (93). In MCMV, m144 fulfills an orthologous mechanism of NK-cell inhibition, mimicking key structural features of the H-2 molecule (94–96). Although its cognate receptor is unknown, both functional and biochemical studies implicate m144 as a key regulator of mouse NK cells (94, 95, 97). Interestingly, UL18 and m144 share more sequence and structure similarities with MHC I molecules than they do with each other, suggesting that convergent evolution led to the independent acquisition of MHC I mimics due to species-specific immune pressure (96, 97) in a way reminiscent of the convergent evolution of iNKR among species.

MCMV m157 is another MHC I-related molecule that may have specifically evolved to engage NK-cell iNKR (98–100). Distinct variations in its three-dimensional folds and the lack of association with β2-microglobulin enables m157 to interact with iNKR with greater affinity than their cognate MHC I ligands (101, 102). Discovery of the inhibitory nature of m157 was made after the initial observations between enhanced viral control and Ly49H stimulatory receptor recognition of m157 (98, 99, 103–106). Despite its dominant activating properties in C57BL/6 (B6) mice and strains bearing Ly49H^b6^, it was hypothesized that m157 initially evolved to inhibit NK cells in the wake of broad downregulations of endogenous MHC I molecules, similar to m144. Indeed, the MCMV-susceptible 129/J allele of the iNKR Ly49I shares substantial sequence similarity with Ly49H^b6^ (107). Moreover, Ly49I^129^ binds to m157-Ig fusion proteins (99) and may even possess a higher affinity for m157 than Ly49H^b6^ (101). Despite the limitations of fully resolving the inhibitory effects of m157 on Ly49I^129^ *in vivo*, it was demonstrated that 129/J mice infected with Δm157 virus exhibited limited, but significant, reductions in viral titers compared to WT BAC-MCMV-infected mice (103). Further support for m157’s immune evasive role can be gleaned from the work of Scalzo and colleagues who found that m157 variants isolated from wild outbred mice bind an array of inhibitory Ly49 with a wide range of affinities while very few of them activate Ly49H (100).

It is important to note that infection and immunity are inextricably linked in an evolutionary arms race (10, 108, 109). While immune surveillance mechanisms represent important targets, successful viral evasion strategies can paradoxically become triggers for immune stimulation. Importantly, scientific findings from models investigating viral evasion and immunity are inherently based on a fixed point in evolution. Although viruses rapidly evolve to evade NK-cell detection, the immune system of a population is also constantly evolving to overcome these hurdles (110).

This observed co-evolution has been independently substantiated by de Boer and colleagues through *in silico* modeling. Using a simplified model of human KIR diversity and a herpesvirus-like agent that could downregulate or promote the expression of decoy MHC I molecules, they demonstrated that viral evasion strategies invariably informed the degree of KIR specificity for HLA, and that the existence of decoy models necessitated by a diverse KIR repertoire to distinguish host ligands from decoys (111). The fact that highly variable and rapidly evolving regions of the genome encode so many iNKR strongly indicates their importance in combating viral infections that are also continually adapting to their hosts.

## Evidence for Licensed-NK-Mediated Viral Control

Many sNKR (e.g., Ly49H and NKp46) have been shown to specifically bind proteins expressed by certain viruses including herpesviruses, influenza, and poxviruses (98, 99, 104, 112–115). This allows NK cells to specifically recognize and eliminate viral pathogens. As an example, human XMEN deficiency, which results in an Mg2+ defect and poor NKG2D expression on NK cells, was overcome with Mg2+ supplementation that led to increased NKG2D expression and enhanced EBV clearance (116). NKG2C is another sNKR frequently expressed by NK-cell subsets. NKG2C+ NK cells preferentially expand after exposure to HCMV (117), potentially indicating specific activation in response to the infection.

Since iNKR bind highly polymorphic MHC I ligands and prevent the lysis of normal, healthy cells, viral modulation of MHC I molecules resulting in a loss of self-MHC elicits iNKR release and lysis of infected cells. While this general recognition mechanism is well established, the contribution of iNKR to specific recognition and clearance of viral infections is less clear. A growing body of literature now documents that iNKR+ NK cells also expand and acquire greater reactivity after viral exposure, which raises additional questions: Is NK-cell subset expansion necessarily linked with specific recognition and lysis of infected target cells? What mechanism(s) can explain increased responsiveness of self-licensed iNKR+ NK cells? Can licensed-NK cells recognize and respond to viral infection in the face of viral evasion? Does a licensed-NK-mediated response to viral infection result in NK-cell memory? These and other important questions are presently unanswered and provide the framework for further review of data alluding to the critical role of licensed-NK cells in viral defense.

### Expansion of iNKR+ NK cells in response to viral infection

Previous studies have documented that certain iNKR+ NK-cell subsets consistently expand after viral exposure (105, 118). Several scenarios may account for this. First, iNKR+ NK subset expansion may be related to non-specific effects associated with broad immune activation or inflammation. Expression of NKR is variegated, but unequal within the NK compartment. Thus, certain highly represented iNKR may simply have an expression advantage and will be primed to expand during NK proliferation. Alternatively, accumulation of iNKR+ NK cells could result from co-expression with an unknown NKR that is required for the specific recognition of and response to infection, essentially making it a biomarker of expansion. Lastly, a given iNKR+ NK subset may increase as the result of the iNKR itself specifically detecting and responding to an infection. Expansion due to specific recognition has been shown for the stimulatory Ly49H receptor and specific recognition by a licensed-NK cell could result in a similar outcome. Importantly, these possibilities are not mutually exclusive, and NK-cell expansion could result from multiple, simultaneous mechanisms.

In B6 mice, expansion of the iNKR Ly49G2+ (G2+) NK-cell subset is a common observation. This G2+ NK-cell expansion appears to occur non-specifically in response to a wide spectrum of conditions, including diverse viral and bacterial infections, sterile stimulation of the immune system, and hematopoietic stem cell transplant (HSCT) (119, 120). However, this is unlikely to be a general feature of iNKR since a propensity for expansion has not been observed with any other inhibitory Ly49+ NK subset. Rather, selective G2^b6^ allelic regulation or epistatic interactions could affect its expression in NK cells. This is supported by studies showing G2^balb^+ NK cells failed to similarly expand after poly I:C treatment or MCMV infection (85, 119, 121). Even in F1 mice expressing both G2^b6^ and G2^balb^ receptors, G2^b6^+ NK cells expanded to a greater degree when directly compared to G2^balb^+ NK cells (119). Further study is needed to clarify the role of G2^b6^+ NK cells in response to conditions that elicit NK-cell expansion and what this propensity for expansion may mean.

On the other hand, G2^c57l^+ NK cells were found to selectively expand and control MCMV infection in the presence of the self-MHC I ligand D^k^. The proportion of G2+ NK cells reliably increases in response to MCMV infection when D^k^ is expressed. However selective increases in G2+ cell number have been most evident in mice given relatively high dose MCMV infection (122). This suggests that the G2^c57l^+ NK cells are uniquely reactive against MCMV targets and, as a result, preferentially accumulate. Extensive genetic analysis has shown that G2+ NK-mediated viral control is evident over a large range of infectious MCMV doses and it is always highly correlated with expression of the self-ligand D^k^ (123, 124). That the increase in NK-cell numbers is dose dependent further supports that this is a specific response to MCMV infection. Two major questions requiring further study arise from these observations: (1) Is the licensed-NK response against MCMV dependent on a threshold level of viral infection? and (2) Does the licensed-NK response cooperate with sNKR (e.g., Ly49P and/or NKG2D) recognition of key targets to mount the observed NK-cell expansion?

Human studies have documented expansion of NK subsets marked by combinations of sNKR and self-specific inhibitory KIR (e.g., NKG2C and iKIR) (16). This feature is prominent in HCMV infection (117, 125–131), but may also occur in hantavirus (132), chikungunya virus (133), HIV-1 (125, 126, 134), and hepatitis infections (135). Unfortunately, extensive co-infection has made it difficult to resolve if the observed NK-cell expansion associated with the latter four is HCMV-dependent. However, a study on EBV and CMV co-infection showed that while all CMV seropositive children had increased NKG2C+ NK cells, the amount of NKG2C skewing was even greater in patients that were CMV/EBV double-seropositive (136). While still inconclusive, this could suggest that the co-infection can increase the NKG2C representation either by providing additional, non-specific effects from EBV infection or that the CMV-experienced NKG2C+ NK may specifically respond to the EBV infection. An important issue to be addressed is whether the self-licensed KIR on the expanded cells are simply endowing the NKG2C+ cells with an enhanced ability to expand or are explicitly contributing to specific viral recognition and NK activation. Additionally, these NKG2C+ cells have been described by some as memory NK (128), which presents the intriguing possibility that iNKR could mark memory NK.

A study by Alter et al. (137) has also reported that KIR3DS1+ and KIR3DL1+ NK cells expand in peripheral blood during HIV infection. However, in related work, only the KIR3DS1+ cells were observed to expand (138). Nonetheless, the expansion depended on the presence of KIR3DL1, and individuals with two copies of KIR3DL1 exhibited a clear enhancement in KIR3DS1 expansion over those with one copy. While this undoubtedly indicates a role for the iNKR, its relationship with the stimulatory receptor is still unclear.

### Specific iNKR responses to viral infections

It was the pioneering work of Scalzo and Shellam, studying genetic control of the host response to MCMV, which led ultimately to the discovery of Ly49H – a major MCMV resistance factor expressed in B6 mice (139). Ly49H binding to MCMV m157 was shown to increase NK-cell proliferation and direct lysis of MCMV-infected target cells (98, 99, 140). Scalzo’s studies further revealed that the B6-related MA/My mouse strain also relies on NK cells to mediate MCMV resistance (141). This result prompted extensive genetic screening for additional host genetic factors that protect against viral infection.

We have used classical genetics approaches to analyze the offspring obtained by crossing MA/My with C57L (MCMV susceptible), another B6-related mouse strain. Because the NKC-Ly49 haplotype common to both strains differs from B6 (142), it was reasoned that either a minor NKR polymorphism or an NKC-independent effect produced the difference in viral control. Using a parallel genetics screen, Vidal and coworkers compared MA/My with the BALB (also MCMV susceptible) mouse strain (143). Both genetic approaches mapped a critical interval centered on the gene coding for MHC I D^k^ (122, 143, 144). Its role in MCMV resistance was verified in C57L-derived transgenic D^k^ mice and in FVB-derived (also MCMV susceptible) class I null mice (123, 145). Together these studies demonstrated that MHC and non-MHC genetic factors regulate NK-mediated MCMV resistance and that the MHC I D^k^ molecule itself has a major effect on viral control.

MHC I D^k^ expression was expected to correspond with enhanced MCMV target detection and lysis via a sNKR, analogous to Ly49H+ control in B6 mice. Ly49P was proposed to mediate MCMV control based on genetic analysis of the resistance phenotype and its recognition of MCMV-gp34-associated D^k^ molecules on infected cells (113, 143). However, its role has been difficult to verify *in vivo* without monospecific mAbs for detection and immunodepletion of Ly49P+ NK cells. NKG2D+ NK cells could also contribute to D^k^-mediated MCMV resistance (85). Despite these possibilities, we found that D^k^-mediated MCMV resistance in MA/My and C57L-derived D^k^+ mice was abrogated by specific immunodepletion of G2+ NK cells. Diminished virus control due to G2+ NK-cell deficiency was observed after treatment with either of two different G2-specific mAbs, which verifies the crucial role of the subset in viral detection and clearance (122, 123).

However, iNKR signaling might also interfere with sNKR activation and viral control. This was examined in neonatal B6 mice adoptively transferred with adult NK cells. MCMV-specific Ly49H+ (H+) NK cells sorted into self-licensed Ly49C/I+ and unlicensed Ly49C/I− NK subsets were assessed for their ability to confer viral control (146). Despite the fact that all H+ cells became activated after MCMV exposure, the unlicensed-NK cells exhibited more effective viral control (146). These data demonstrated that unlicensed-NK cells could have a dominant role in viral clearance, particularly when viruses successfully escape licensed-NK cells.

Although MCMV evaded and perhaps even exploited self-licensed-NK-cell detection in the above B6 mice, differences in the different models of NK-mediated viral immunity suggest that licensed-NK-mediated viral control may depend on specific recognition of virus-infected targets via altered MHC I sensing. This question was further tested in a large-scale genetic analysis of the host response to MCMV (124). More than 200 mice disparate for the G2^c57l^ cognate-ligand, D^k^, were analyzed for immune and NK-cell features in the response to infection, in parallel with viral control. Remarkably, the results demonstrated that G2 expression on NK cells, the percentage of G2+ NK cells before infection and their expansion afterward, in addition to MCMV resistance, were all linked to the presence of D^k^ in the genome. We infer from these results that self-D^k^-licensed-NK cells were effectively poised to efficiently recognize and respond to targets, which led to more effective sNKR signaling, lysis of infected cells, and viral control.

The results above predict that licensed-NK cells may also contribute viral control in B6 mice, as long as one or more iNKR adequately recognize altered expression of a key self-ligand. In agreement with this, Murphy and colleagues reported that licensed-NK cells selectively expanded following MCMV exposure in syngeneic HSCT recipients, which corresponded to increased viral control (147). A similar result was observed in allo-HSCT recipients, which implies that licensed-NK cells were generally more responsive to MCMV in transplanted recipients (148). An intriguing possibility is that a licensed-NK response to MCMV is actively suppressed by regulatory T cells and TGFβ in B6 mice, but becomes measurable after HSCT and in T_reg_- or TGFβ-depleted mice (147). Though this seems inconsistent with MCMV gp34-mediated interference with iNKR detection, it may be possible that gp34 stabilization of critical self-ligands is less effective in the setting of HSCT. Indeed, NK-cell reactivity is sensitive to MHC I expression after HSCT (149, 150), and the functional reactivity of licensed-NK cells in HSCT patients was found to be responsive to self-ligand expression on donor-derived hematopoietic cells (i.e., NK are functionally licensed on transplanted donor cells following HSCT) (151). Further work is needed to fully understand the genetic and environmental impacts on NK-cell licensing and reactivity and how these influence NK behavior during infection.

Human studies also provide evidence that individuals with a given configuration of matched iNKR:HLA class I may exhibit enhanced viral control. Correlations between matched inhibitory KIR and HLA have been observed in responses to HIV-1 (3DL1:HLA-Bw4), HCV (2DL3:HLA-C1), influenza (3DL1:HLA-Bw4; 2DL2/DL3:HLA-C1), and vaccinia virus (VV) (NKG2A:HLA-E) [(152); reviewed in Ref. (16, 153)]. These associations provide evidence for a direct contribution of iNKR and licensed-NK cells to virus control.

As discussed above, 3DL1/S1+ NK cells can expand during HIV-1 infection. This expansion is dependent on the expression of the cognate-ligand for these KIR in the individual (HLA-Bw4-80I) (137, 138). Individuals who express this particular HLA exhibit better control of HIV viremia as well as significant protection from developing AIDS (67). In a group of controllers expressing the 3DL1:HLA-Bw4 pair, protection was more strongly associated with high NK-cell activity than CD8 T cell responses (69). HIV-1 peptide antagonism, in part, may account for these observations: the HLA-Bw4 restricted immunodominant Gag240–249 TW10 T cell epitope is targeted by CD8+ T cell effectors early after infection. HIV-1 variants of the TW10 epitope, while successful at evading CD8+ T cells, often interfere with 3DL1 binding to its cognate-ligand and thereby potentially render infected cells more susceptible to “missing-self” detection by NK cells (154). In addition, expression of increasing levels of strongly inhibitory 3DL1 in conjunction with its ligand enhances the observed protective effect of this iNKR, further strengthening the link between this KIR:HLA pair and HIV control (68).

Genetics studies have also indicated a potential role for HLA-C (alleles of which can serve as ligands for 2DL1, 2DL2, and 2DL3) expression in HIV control, but the importance of NK cells and KIR:HLA pairing in this observation is still uncertain (155–160). Some work has indicated that 2DL2 exerts significant pressure on HIV. Individuals with this iNKR have a prevalence of HIV-polymorphisms in a region spanning the Vpu and Env proteins. These polymorphisms allow binding of 2DL2 upon HLA-C presentation whereas the HIV wild type sequences do not (161). In a related study screening 217 different Gag peptide sequences, only 11 were found to stabilize HLA-Cw-0102, and of those 11, only one of these could mediate inhibition of 2DL2 expressing NK cells (162). These studies suggest that, even though HLA-C is not modulated by Nef on the surface of infected cells, many HIV peptides are inefficient at inhibiting 2DL2+ NK cells, pressuring the virus to rely on peptide diversification during infection of individuals with 2DL2.

Natural killer cells are generally activated in response to HCV infection (when compared with NK from uninfected controls), but NK cells positive for 2DL2/L3 tended to exhibit increased degranulation in response to K562 targets; particularly in cases of self-limited infection (163). Individuals homozygous for the 2DL3:HLA-C1 pair exhibit enhanced control of the virus following low-dose exposure and this partnership is also associated with spontaneous clearance of the infection (164, 165). In addition, the homozygous genotype is correlated with apparent resistance to infection in i.v. drug users (prolonged seronegativity despite continual exposure) and positive responses to treatment after HCV infection (166).

In a model of human influenza A virus (IAV) infection, NK cells from subjects homozygous for 2DL3 and HLA-C1 were more reactive to IAV infected cells than those from individuals homozygous for the 2DL1:HLA-C2 ligand pair (167). Another study looked at the influence of 3DL1, 2DL1, and 2DL2/3 in H1N1/09 infected ICU patients (2009 pandemic IAV strain). The results showed that individuals expressing all three iNKR had the lowest risk of death and that ICU patients exhibited decreased ligation of 3DL1/S1 by HLA-Bw4 and 2DL1 by HLA-C2 (168), suggesting that stronger interactions between these KIR:HLA pairs may be protective on some level.

While the human NK response to VV infection appears to involve NK cells to some degree, the role of iNKR:HLA has not been rigorously studied. However, an *in vitro* investigation using an autologous cell system showed differences in VV reactivity among NK subsets from an individual subject. NK cells expressing high levels of NKG2A were far more efficient at lysing VV infected cells than their 2DL1+ or NKG2A^dim^ counterparts (152). This increased lysis was abrogated by rescuing HLA-E expression on target cells, indicating the activity was due to NKG2A-mediated detection of reduced HLA-E.

**Figure 3:**
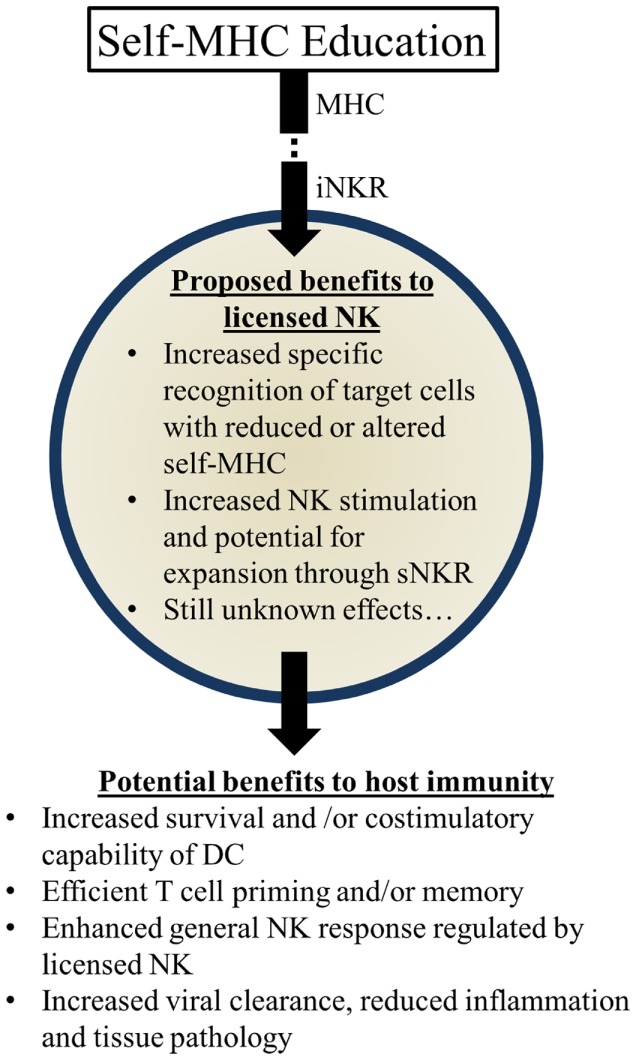
**Summary of proposed beneficial effects of licensed-NK cells**. NK Licensing potentially impacts the immune response in a variety of ways. Benefits may manifest directly as NK specific recognition or indirectly via effects on their environment and other cells.

Several possibilities could account for the apparent benefits of these genetic pairings: (1) KIR:HLA pairings may correspond with more effective NK-mediated viral control. Early control of viral replication and/or less severe inflammation may correspond to less morbidity and mortality over time. (2) Genetic pairings favoring NKR and HLA could affect NK-mediated regulation of adaptive immune responses. More efficient priming of T cells and less interference with T cell mediated immunity could substantially augment viral control and clearance. (3) Genetic pairings might affect other immune cell features (e.g., DC numbers and/or costimulatory molecule expression) that have the potential to affect NK cells and/or T cells needed to further control viral infection and replication. (4) Licensed-NK cells may regulate other types of NK cells, including unlicensed-NK cells, to mount a more vigorous attack against infected targets. We speculate that iNKR-mediated sensing of viral targets can enhance the responsiveness of NK cells to stimulatory receptor signaling and augment their capacity to lyse infected targets. It will be important to rigorously investigate these possibilities in the future and identify the mechanisms through which iNKR-licensed-NK cells can augment immunity and virus control (Figure 3).

## Closing Remarks

Important insight into the benefits of iNKR-mediated enhancement of NK-cell reactivity has been gleaned from analyzing NK-cell responses during viral infection. It is known that iNKR tune NK cells to efficiently detect and lyse target cells that lack self-MHC I expression, including virus-infected cells. Viral immune evasion strategies that target iNKR+ NK cells lend credence to their importance in defending against viral infection. The value of iNKR in immunity and survival is also evidenced by their “conserved diversity.” This trait is reminiscent of MHC polymorphism shaped by co-evolution with diverse pathogens. It is clear that NK-cell responses are intimately tied to the expression and diversity of iNKR and MHC I. Ultimately, the receptor–ligand interactions between these two protein families can shape NK-mediated immunity, host protection, and evolutionary success. Further study of iNKR in viral immunity should yield better understanding of NK-cell education and its functional role in viral resistance. Learning more about iNKR and the regulation of NK cells should also help in realizing their potential for clinical applications in chronic infection, transplantation, tumor immune therapy, and/or immune deficiency.

## Conflict of Interest Statement

The authors declare that the research was conducted in the absence of any commercial or financial relationships that could be construed as a potential conflict of interest.
